# Japan-Medical Image Database (J-MID): Medical Big Data Supporting Data Science

**DOI:** 10.14789/ejmj.JMJ25-0004-P

**Published:** 2025-06-04

**Authors:** TOSHIAKI AKASHI, KANAKO K. KUMAMARU, AKIHIKO WADA, MASAHIRO HASHIMOTO, KENJI HIRATA, YAYOI HAYAKAWA, KATSUHIRO SANO, KOJI KAMAGATA, AKIFUMI HAGIWARA, YUTAKA IKENOUCHI, SHIGEKI AOKI

**Affiliations:** 1 Department of Radiology, Juntendo University School of Medicine, Tokyo, Japan; 1 Department of Radiology, Juntendo University School of Medicine, Tokyo, Japan; 2 Department of Radiology, Keio University School of Medicine, Tokyo, Japan; 2 Department of Radiology, Keio University School of Medicine, Tokyo, Japan; 3 Department of Diagnostic Imaging, Faculty of Medicine, Hokkaido University, Sapporo, Japan; 3 Department of Diagnostic Imaging, Faculty of Medicine, Hokkaido University, Sapporo, Japan; 4 Faculty of Health Data Science, Juntendo University, Chiba, Japan; 4 Faculty of Health Data Science, Juntendo University, Chiba, Japan

**Keywords:** database, medical images, DICOM, radiology

## Abstract

The digitization of radiology practices has advanced for nearly two decades, exemplified by the global standard Digital Imaging and Communications in Medicine (DICOM). Rapid technological progress in imaging modalities has led to a significant increase in the volume of data handled. However, it has become difficult for the limited number of radiologists in Japan to maintain the quality of diagnosis while efficiently processing the data. In response, the Japan Radiological Society (JRS) advocated the “Japan Safe Radiology” Initiative, which aims to improve the safety, efficiency, and quality of radiological medicine by actively utilizing information and communication technology (ICT) in all aspects of radiological practice.

Recent advances in innovative artificial intelligence (AI) technology have shown a high affinity for image processing, prompting recognition of the importance of using big data systems to integrate radiological medicine. Consequently, in 2017, the Japan Agency for Medical Research and Development (AMED) supported the JRS project, Development Research for the Realization of a National Image Diagnosis Database, through which the Japan Medical Image Database (J-MID) was established.

The J-MID is designed to centralize medical resources and systematically collect CT/MR images and diagnostic reports from 10 major university hospitals in Japan through an academic information network (SINET). Data were anonymized and stored on a central server in the cloud, enabling researchers to utilize J-MID conveniently. In April 2024, J-MID had collected more than 534 million images (1.65 million cases), making it an unparalleled repository of real-world radiological data in Japan.

## Background of the development of the J-MID

The digitization of radiology practice has advanced for nearly two decades, transitioning from film-based to monitor-based diagnosis, as exemplified by the global standard Digital Imaging and Communications in Medicine (DICOM). Rapid technological progress in imaging modalities has led to a significant increase in the volume of data handled. However, it has become increasingly difficult for a limited number of radiologists to maintain diagnostic accuracy while efficiently processing data. In response, the Japan Radiological Society (JRS) advocated the “Japan Safe Radiology” initiative, which aims to consistently improve the safety, efficiency, and quality of radiological medicine by actively utilizing Information and Communication Technology (ICT) in all aspects of radiological practice, including imaging equipment, examination orders, examinations, and image interpretation.

Recent advances in innovative artificial intelligence (AI) technology have shown a high affinity for image processing, prompting recognition of the importance of using big data systems to integrate radiological medicine. Consequently, in 2017, the Japan Agency for Medical Research and Development (AMED) supported the JRS project “Development Research on the Establishment of a National Database of Medical Diagnostic Images,” through which the Japan Medical Imaging Database (J-MID) was established in March 2018. This research (E21-0099) was approved by Research Ethics Committee, Faculty of Medicine, Juntendo University.

## Organization of the J-MID

The J-MID is designed to centralize the medical resources located in each hospital and systematically collect DICOM data of CT images and their diagnostic reports from seven major universities (Juntendo University, University of Tokyo, Keio University, Osaka University, Okayama University, Kyoto University, and Kyushu University) via the academic information network (SINET). Since 2020, the database has expanded to include MR images and radiation dose-structured reports (RDSRs).

Data were anonymized at each hospital using the unique J-MID method and automatically sent to a central on-premise server at Kyushu University. Anonymization of DICOM metadata was based on a guideline, ‘Anonymization Technique Guide for Image Medical Systems' edited by the Japan Medical Imaging and Radiological Systems Industries Association^1)^. Regarding the anonymization of patient ID, considering future collaboration with other medical data, we adopted the common method used for ID hashing in the Japan Excellence for Diagnostic Imaging^2)^.

There were several difficulties in managing on- premise servers. Connection and communication to the J-MID server at Kyusyu University were challenging for members other than those at Kyusyu University. For example, it took approximately three days to download all images acquired daily in all hospitals from the J-MID server because of slow DICOM communication with the center server. J-MID was inconvenient for researchers because of the limited access to terminals that could be connected to the J-MID server to ensure security.

**Figure 1:**
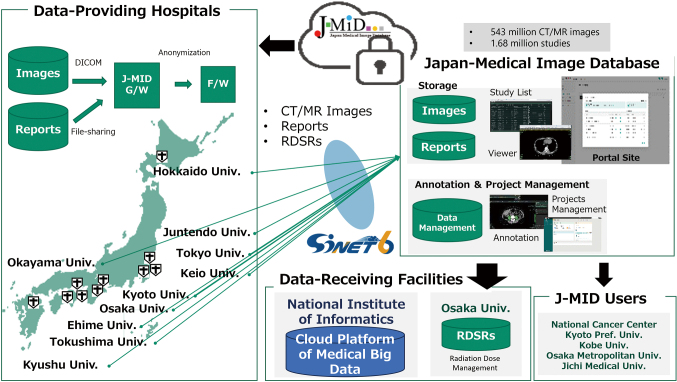
Outline of the J-MID infrastructure system J-MID collects medical images and reports of computed tomography (CT) and magnetic resonance imaging (MR) from 10 university hospitals in Japan, and data are sent to a center server on the cloud after anonymization. Five other facilities have user accounts for the J-MID cloud. Researchers can browse, search, and download data by connecting to the J-MID portal site online. J-MID also has a platform for project management, including an annotation tool. Data are provided to the ‘Cloud Platform of Medical Big Data’ operated by the National Institute of Informatics, and data of radiation dose structured reports are sent to Osaka University.

The equipment of the system should be renewed periodically at the end of each service life. While developing J-MID, we considered a cloud server to save costs, especially for equipment renewal. However, in a proof-of-concept study of the J-MID construction in the cloud in 2019, we decided not to move to the cloud because of security concerns and high maintenance costs. At that time, the ‘Guidelines for Building Information Systems Used in Medical Image Data Collection Projects' published by the Japan association for medical informatics recommended establishing a demilitarized zone (DMZ) and installing a gateway device within this zone to mediate between the VPN space and the DMZ environment^3)^. However, modifying each university's servers to comply with these guidelines presented significant implementation challenges. Since AMED's research support ended in April 2022, JRS has inherited and managed the J-MID system. Three universities (Ehime University, Hokkaido University, and Ehime University) joined J-MID as data-providing facilities, which will now consist of 10 university hospitals since 2023 (Figure 1).

## Systems of the J-MID on the cloud

In April 2023, the on-premises J-MID server at Kyushu University was abandoned, and its data were migrated to the Medical Cloud Service (FUJIFILM Medical, Tokyo, Japan). Simultaneously, the transmission gateway servers were renewed at each hospital. Anonymizing DICOM metadata and building a database were also considered to improve searchability. By connecting to the J-MID portal site on the cloud via the internet, researchers can browse, search, and download data from images and reports. Moving the server to the cloud improved the accessibility to J-MID and promoted research activities.

**Figure 2:**
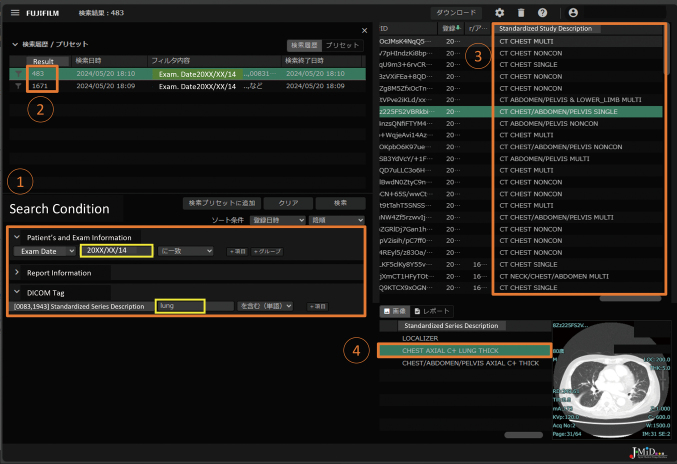
Study list of J-MID This was the search screen for the J-MID study list. 1,2) 1,671 studies were conducted at ‘20XX/XX/14’ in 10 hospitals. Among them, 483 series included ‘lung’ in ‘Standardized Series Description’. 3) A result list includes ‘CHEST’ in the ‘Standardized Study Description’ as a body part of the imaging. 4) A series of lung window images with the standardized series description of ‘CHEST AXIAL C+ LUNG THICK’ is displayed in a study of ‘CT CHEST/ABDOMEN/PELVIS SINGLE.’

**Figure 3:**
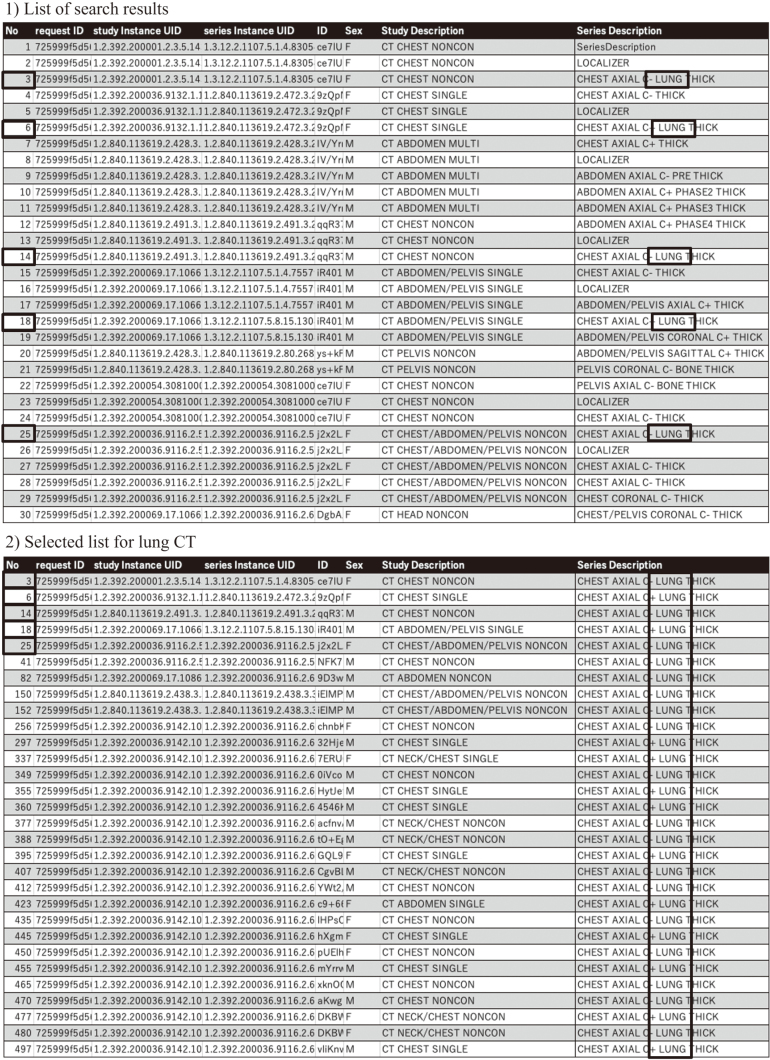
New file-download system 1) Creating search results in CSV file format is possible on the J-MID cloud. 2) After data cleansing the list to select the series of CT images with a lung window (for example), the selected series within the list can be obtained by uploading the list to the J-MID system.

DICOM metadata must be stored in a database to search for images required for specific purposes. Several DICOM attribute names, such as study description and series description, are essential for identifying the types of images related to radiological acquisition techniques. However, because these attribute names were not unified in every hospital and were anonymized in the J-MID, we could not identify the types of images in the J-MID. To solve this problem, we introduced the Curie ENDEX application (Enlitic, Colorado, USA) to the cloud in November 2023 to determine relevant clinical information using natural language processing and by looking at medical images and their associated DICOM metadata. DICOM attribute names, including ‘study description’ and ‘series description’ standardized by the application, are also stored in the J-MID and can be searched on the portal site (Figure 2). We have also built a new file download system on the cloud. It is also possible to create search results in the CSV file format and obtain only the required cases by uploading the selected list to the download tool of the J-MID cloud after data cleansing (Figure 3).

**Figure 4:**
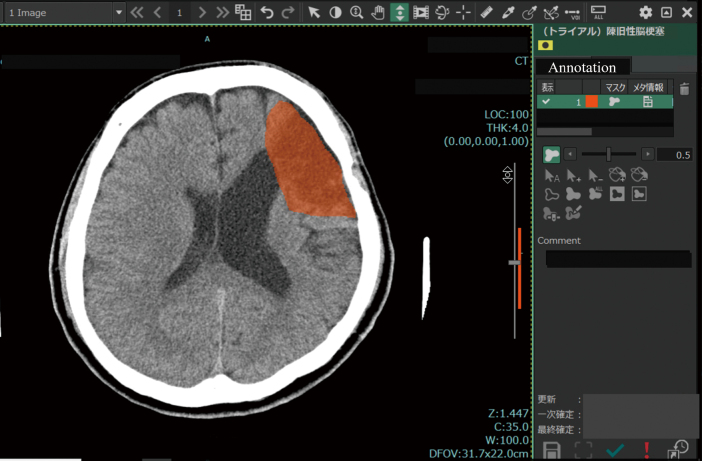
Annotation tool to draw the 2D region of interest (ROI) The old infarction was annotated with orange ROI.

**Figure 5:**
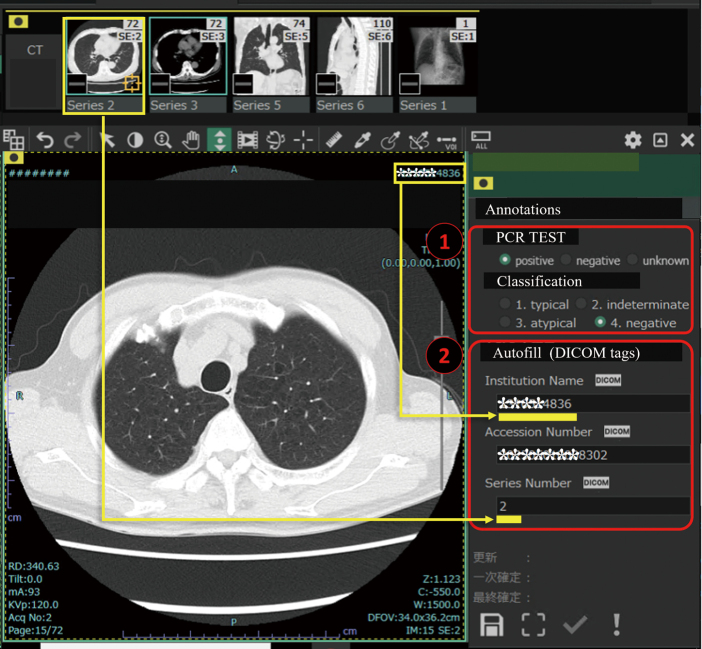
Annotation tool for the classification of COVID-19 pneumonia 1) The results of the PCR test and the classification diagnosed by a radiologist can be selected using radio buttons. 2) Selected DICOM attribute names (institutional name, accession number, and series number) were attached automatically to the annotation data.

The J-MID has a platform ‘SYNAPSE Creative Space’ (FUJIFILM Medical, Tokyo, Japan), for project management that includes an annotation tool on the cloud. A project administrator can assign cases selected from a database to annotators and create a template for annotation to obtain an appropriate type of annotation for the project. Annotation tools (Figure 4, 5) can create 2D or 3D annotations and classifications based on text or numbers. DICOM metadata can also be automatically attached to the annotation by selecting DICOM tags such as series number (0020,0012), institution name (0008, 0080), and accession number (0008, 0050). These applications can be used by anyone with an account on the platform using a remote desktop connection over the internet.

However, owing to the anonymization of patient IDs on the gateway server in each hospital, cases that should be annotated can no longer be identified on the J-MID cloud. It is necessary to modify the system to resolve this problem by registering patient IDs in each hospital prior to anonymization.

## Collaboration with the J-MID

The J-MID provides data to the ‘Cloud Platform of Medical Big Data’ operated by the National Institute of Informatics (NII; Tokyo, Japan) as a platform for AI technology for analyzing medical images and cloud-based platform design for medical imaging big data using the SINET academic information network, designed and operated by NII^4)^. This project was within AMED's “Program on ICT Infrastructure Development for Clinical Research” under the theme “Research on the design of database platforms for diagnostic images and other data to facilitate the use of information and communication technologies and methods of artificial intelligence.”

From April to June 2020, during the early initial phase of the epidemic, we collected 368 chest CT studies of polymerase chain reaction (PCR)-positive COVID-19 pneumonia. Annotations were made according to the radiological criteria of the RSNA^5)^. These data were shared with computer engineering researchers at the NII and collaborative institutions throughout Japan. They delineated institutional responsibilities and developed an AI model capable of predicting the typicality of COVID-19 pneumonia with an accuracy of 83.3%^6)^. This proves that the J-MID system works well, even in critical situations. We also conducted a proof-of-concept study of CT surveillance for COVID-19 pneumonia from 2021 to 2022 using the J-MID and the NII cloud platform with the NII and Nagoya University because CT surveillance on J-MID was considered helpful in detecting COVID-19 pneumonia in Japan^6)^. In this study, 310,000 chest CT scans were analyzed for surveillance. Automatic selection of chest CT images reconstructed for the lungs from all CT studies was recognized as more difficult than expected. For quick searches, the database should be organized appropriately by classifying images according to body parts, contrast enhancement, acquisition protocols, etc. Pre-processing is required to eliminate unnecessary data and save computational resources for effective surveillance. Moreover, image analysis should be performed simultaneously retrospectively and prospectively to identify changes after the occurrence of diseases. It is also understood that the database must have preserved pre-occurrence data for future use.

The National Cancer Center, Kobe University, the Kyoto Prefectural University of Medicine, Jichi Medical University, and Osaka Metropolitan University have become JMID users. The J-MID comprises 10 data-provider hospitals and 15 data-receiving facilities from six hospitals and nine engineering departments. In May 2024, J-MID collected more than 543 million images (1.68 million cases).

## Conclusions

J-MID continues to collect medical images and radiological reports from 10 university hospitals, and the data are sent to the J-MID center server in the cloud. J-MID has become an unparalleled repository of real-world radiological data in Japan and has developed a platform for managing research in the cloud to improve its usage.

## Funding

This study was supported by JSPS KAKENHI (grant number 22K10535) and Cross-ministerial Strategic Innovation Promotion Program (SIP) on “Integrated Health Care System” (grant number JPJ012425).

## Author contributions

TA was a major contributor in writing the manuscript. TA, KKK, AW, MH, KH, KH, KS, KA, YH, AH, and YI contributed to the construction of the new J-MID system. SA was a supervisor of this research. All authors read and approved the final manuscript.

## Conflicts of interest statement

The authors declare that there are no conflicts of interest.

## Data availability

Not applicable.
